# fMRI and Brain Activation after Sport Concussion: A Tale of Two Cases

**DOI:** 10.3389/fneur.2014.00046

**Published:** 2014-04-14

**Authors:** Michael G. Hutchison, Tom A. Schweizer, Fred Tam, Simon J. Graham, Paul Comper

**Affiliations:** ^1^Faculty of Kinesiology and Physical Education, University of Toronto, Toronto, ON, Canada; ^2^Keenan Research Centre for Biomedical Science of St. Michael’s Hospital, Toronto, ON, Canada; ^3^Faculty of Medicine, University of Toronto, Toronto, ON, Canada; ^4^Physical Sciences, Sunnybrook Research Institute, Toronto, ON, Canada

**Keywords:** concussion, sport, mild traumatic brain injury, fMRI, brain imaging

## Abstract

Sport-related concussions are now recognized as a major public health concern: the number of participants in sport and recreation is growing, possibly playing their games faster, and there is heightened public awareness of injuries to some high-profile athletes. However, many clinicians still rely on subjective symptom reports for the clinical determination of recovery. Relying on subjective symptom reports can be problematic, as it has been shown that some concussed athletes may downplay their symptoms. The use of neuropsychological (NP) testing has enabled clinicians to measure the effects and extent of impairment following concussion more precisely, providing more objective metrics for determining recovery. Nevertheless, there is a remaining concern that brain abnormalities may exist beyond the point at which individuals achieve recovery in self-reported symptoms and cognition measured by NP testing. Our understanding of brain recovery after concussion is important, not only from a neuroscience perspective, but also from the perspective of clinical decision-making for safe return-to-play. A number of advanced neuroimaging tools, including blood oxygen level dependent functional magnetic resonance imaging (fMRI), have independently yielded early information on abnormal brain functioning. In the two cases presented in this article, we report contrasting brain activation patterns and recovery profiles using fMRI. Importantly, fMRI was conducted using adapted versions of the most sensitive computerized NP tests administered in our current clinical practice to determine impairments and recovery after sport-related concussion. One of the cases is consistent with the concept of lagging brain recovery.

## Introduction

### Case 1

JZ is a 20-year-old male varsity hockey player who sustained a concussion on November 25 while participating in a game. The injury occurred as a result of contact with an opponent where he was struck by the player’s gloved hand to the right side of the head. There was no loss of consciousness or amnesia associated with the event, although he reported increased pressure to the right parietal area, headache, and felt “in a fog”. JZ was referred to a sport medicine physician for follow-up.

#### MD first office visit

Four days post-injury, a sport medicine physician evaluated JZ. JZ reported one previous sport-related concussion with no residual complaints. At the time of his medical assessment, a standard brief neurological assessment that included orientation, speech, gait, balance, coordination, and peripheral motor and sensory function, was normal. Cranial nerve examination included smooth pursuits and vergence facility in extra-ocular movements, and Dix–Hallpike test to attempt to provoke positional nystagmus. No abnormalities were identified during the general neck assessment. At this time, a total symptom score of 3 was reported on the University of Toronto concussion symptom scales (UTCSS) with mild symptoms of difficulty concentrating, difficulty reading, and feeling “off” or “not normal”/in a “fog.” The UTCSS is a symptom scale analogous to other symptom scales which provides a formal method of documenting post-concussion symptoms (immediately after injury or at any time thereafter). The total symptom “score” is the sum of 17 symptom ratings (min score = 0; max = 68). We have shown that the UTCSS is a valid tool for use in clinical and research settings, and previous research indicates an average score for healthy, uninjured athletes is approximately a total score of 4 (1). The standardized firm surface balance error scoring system (BESS) was completed, with seven errors documented (single leg = 2; tandem = 5) for a score of 23/30.

#### Neuropsychology consultation

All athletes participating in the institution’s varsity sports teams complete preseason computerized neuropsychological (NP) screening prior to their first year of participation (i.e., a “baseline” assessment). If an athlete suffers a concussion, they then complete post-injury NP testing at the time of a consultation with the neuropsychologist. The NP assessment and consultation occurs prior to medical clearance once the athlete successfully completes exertion progression, analogous to Step 5 in the most recent concussion in sport consensus return-to-play (RTP) guidelines (2). At the time of the NP consultation (December 15), JZ reported no cognitive complaints and no history of significant medical difficulties/disorders/diseases, early childhood or developmental difficulties, mental health difficulties, alcohol/substance use issues, attention deficit disorder, learning disorder, or behavioral problems. His scores on the Automated Neuropsychological Assessment Metrics (ANAM) battery were compared to a normative database for male athletes in his age group as well as his baseline test scores. Overall, the mood profile was normal (i.e., low negative mood states and elevated positive mood states). With respect to cognitive functioning, indices of mean reaction time, accuracy, and a global efficiency index of accuracy and speed (i.e., throughput) were evaluated. All ANAM subtest scores were above the 15th percentile and not significantly declined from baseline assessment. Therefore, the opinion of the neuropsychologist was that there was no definitive change in neurocognitive status that prevented JZ from returning to full sports participation.

#### Outcome

On December 2, JZ underwent functional MRI assessment for research purposes [functional magnetic resonance imaging (fMRI) results described below] and results were not considered in the medical clearance decision process. JZ was medically cleared to return to full sport participation on December 16.

### Case 2

HC, a first year male volleyball player (19 years old), sustained a concussion during a team practice on October 27. HC was struck on the back of his head by a teammate’s elbow and fell to the gymnasium floor, but did not strike his head on the floor. No loss of consciousness or amnesia was associated with the event; but he reported feeling dazed, headache, and that “lights seemed dimmer” after the collision with his teammate. HC was removed from practice and required to follow-up with a sport medicine physician.

#### MD first office visit

Four days post-injury, HC reported symptoms of difficulty remembering, feeling slowed down, and fatigue. Consistent with Case 1, the brief neurological (i.e., orientation, speech, gait, balance, coordination, peripheral motor and sensory function, smooth pursuits, vergence facility, and Dix–Hallpike) and general neck exam were normal for HC. A total symptom score of 4 was reported on the UTCSS. Standardized assessment with firm BESS stances resulted in a score of 28/30 (errors: single leg = 1; tandem = 1). HC reported no residual complaints related to one previous concussion (4 years previously), which included a brief loss of consciousness (a few seconds) and possible amnesia.

#### NP consultation

HC completed a baseline computerized cognitive screening test (ANAM) before the athletic season (October 7). At the time of NP consultation, HC reported no history of significant medical difficulties/disorders/diseases, early childhood or developmental difficulties, mental health difficulties, alcohol/substance use issues, attention deficit disorder, learning disorder, or behavioral problems. His ANAM mood scores at the time of consultation were considered normal, with low levels of anxiety, restlessness, fatigue, depression, and anger; as well as elevated levels of vigor and happiness. HC had no post-injury cognitive complaints and all of the cognitive ANAM subtests placed above the 25th percentile compared to age–sex matched peers, without significant decline from his baseline assessment. The opinion of the neuropsychologist was that there was no definitive change in post-injury cognitive status and therefore no neurocognitive reason for HC to be withheld from full participation.

#### Outcome

On November 4, HC underwent functional MRI assessment for research purposes, described below. Similar to Case 1, fMRI results were not included in the medical clearance decision process. HC was medically cleared to resume full sport participation on November 17.

## Background

Although concussions as a neurological phenomenon have been recognized since antiquity, it is only in the past two decades that there has been intense public and scientific scrutiny of the injury. Of particular concern is the substantial evidence pointing to an increased relative risk of sustaining additional concussions after having sustained one concussion, and that this increased relative risk is long-term in nature and perhaps permanent (3–8). Compounding the issue is the risk of persistent cognitive deficits and/or persistent non-cognitive symptoms such as headaches, functional difficulties, and emotional disturbance, which likely increases with the number of concussions (9–14).

Physicians often rely exclusively on subjective symptoms as the indicator of recovery. With limited clinical tools and an inability to depend completely on athletes’ self-report of concussive symptoms, the use of NP score profiles to help assess dysfunction associated with concussion has increased in recent years. A number of computerized NP testing programs are available and these tools are now widely used in elite sport environments, and are rapidly becoming more widespread at the community level. While it is accepted that NP tests alone are not adequate to confirm diagnosis, they are widely used in the rehabilitation and RTP setting as part of an overall concussion evaluation protocol. In this context, NP tests provide an objective assessment of cognitive functioning to ensure that the athlete does not RTP with an increased risk of further injury or delayed recovery. A number of studies have shown the added value of computerized NP testing in the management of sport-related concussion (15–19). These studies show that the sole reliance on symptom reporting is insufficient in determining medical clearance to resume unrestricted sport participation, as there is a tendency for athletes to underreport symptoms (20–22), and/or recovery of symptoms can precede cognitive recovery on computerized testing (16, 23, 24). ANAM is one of a number of computerized NP test batteries used in concussion assessment and management, and research with ANAM has demonstrated accuracy and reliability in the evaluation of sport-related concussion (24–28).

More recently, novel non-invasive techniques have been applied in an effort to provide greater insight into brain functioning following concussion than previously capable. To date, functional MRI has been most extensively evaluated, and several studies have reported functional changes associated with concussion (29–34). Functional MRI typically offers spatial resolution on the order of a few millimeters and thus is well suited to localizing sites of neural activity and potential dysfunction after concussion (35). A key limitation of the literature is that the types of cognitive tests used in previous functional MRI studies have not represented the clinical NP tests administered to determine recovery and to justify RTP following concussion. In response to the gaps in existing knowledge, the fMRI protocol introduced for research purposes in both cases consisted of three adapted ANAM tests (simple reaction task, spatial processing task, and match-to-sample task) to facilitate analysis of the blood oxygen level dependent (BOLD) signal. These tests were designed to assess various cognitive domains such as visuomotor processing, attention, visual spatial skills, and working memory. The fMRI protocol was used to provide *in vivo* measurements of brain changes associated with performance following concussion. The study was approved by the research ethics boards of St. Michael’s Hospital and Sunnybrook Health Sciences Centre, and both athletes gave written informed consent. Imaging was conducted at 3.0 T on a GE MR750 MRI system (GE Healthcare, Waukesha, WI, USA). Structural imaging consisted of a 3D T1-weighted high-resolution anatomical reference scan. Functional imaging used T2*-weighted single-shot spiral-in/out imaging (36) (FOV = 20 cm, matrix = 64 × 64, 5-mm axial slices, TE/TR = 30/2000 ms, flip angle = 70°). Images were analyzed offline using analysis of functional neuroimages (AFNI) freeware (37). Preprocessing included corrections for motion, physiological effects, and slice timing, as well as spatial smoothing (6 mm). Statistical parametric maps of brain activity were generated using a general linear model including boxcar waveforms describing task onset/offset, convolved with a canonical hemodynamic response function. The individual *t*-maps for each of the fMRI NP tasks were spatially transformed into Talairach stereotaxic brain atlas space, and regions of statistically significant brain activity (corrected *q* < 0.05) were thresholded using a False Discovery Method (38).

In summary, we provide two case studies where fMRI was completed 10 days following injury. Brain activity was assessed for functional tasks using a battery of the most sensitive computerized NP tests available, similar to those administered in current clinical practice to determine impairments and recovery after concussion.

## Discussion

With the increased prevalence of sports concussion, and the potentially enduring effects that have been correlated with repeated concussions (39, 40), novel assessment tools – such as neuroimaging and electrophysiological biomarkers – are needed to assist us in better understanding the mechanisms associated with concussion and the recovery process. The above cases illustrate differing brain activation patterns identified by BOLD fMRI (41) in the acute phase of recovery, despite similar clinical presentation and outcome (see Table 1).

**Table 1:** **Summary of clinical cases**.

	Case 1 – JZ	Case 2 – HC
Sport	Hockey	Volleyball
*MD first office visit*		
Memory of events before trauma	Full recall	Full recall
Memory of events after trauma	Full recall	Full recall
Loss of consciousness	None	None
Months of year in reverse order (s, errors)	15, 0	15, 0
Serial subtraction: 100 by 7 s (s, errors)	32, 0	Not administered
Recall three words (number correct)	3	3
BESS (firm surface, out of 30)	23	28
Symptoms	Difficulty in concentrating	Difficulty in remembering
	Difficulty in reading	Slowed down
	Feeling “off” / “in a fog”	Fatigue
Symptom total score	3	4
Days out from sport	20	23

**Figure 1:**
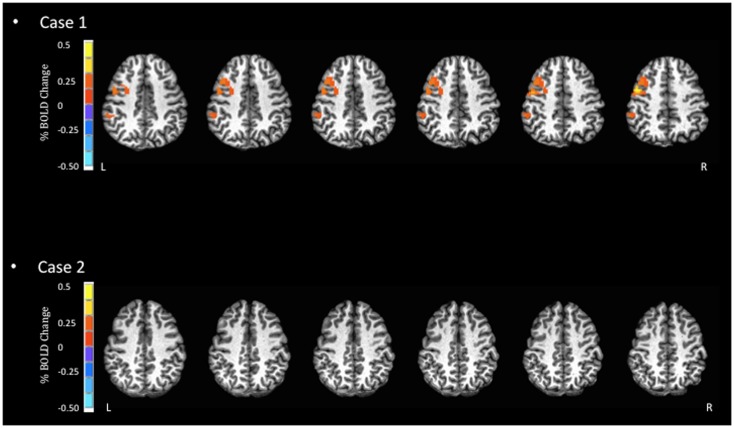
**Blood oxygen level dependent signal activation for simple reaction task**. In this task, the expected BOLD activation should occur in areas such as the pre- and post-central gyri. However, minimal activation is observed for Case 2 in these areas.

**Figure 2:**
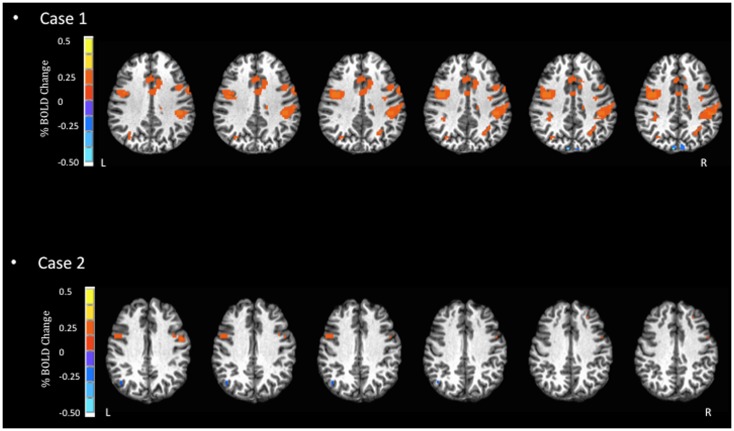
**Blood oxygen level dependent signal activation for spatial processing task**. In this task, Case 1 displays more dispersed and increased BOLD activation in the inferior frontal and pre-central gyri, as well as the insula. In contrast, Case 2 displays little BOLD activation.

**Figure 3:**
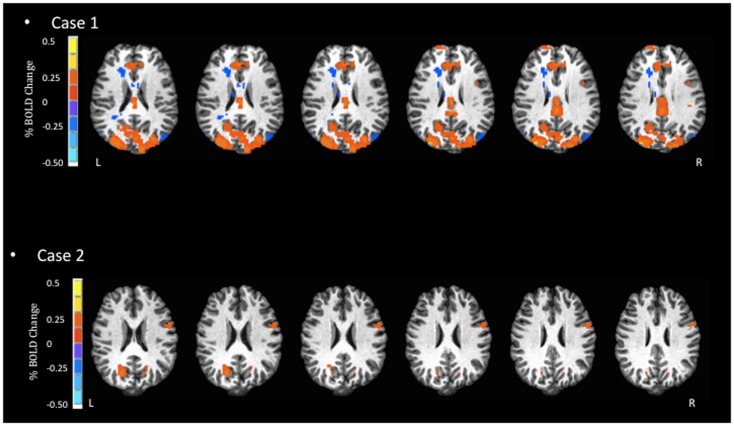
**Blood oxygen level dependent signal activation for match-to-sample task**. In the most complex task, Case 1 has substantially larger BOLD activation compared to Case 2, in the areas of the occipital cortex, paracentral lobule, and anterior cingulate.

A recent review of the relationship between fMRI and mTBI (35) indicated that future fMRI work would undoubtedly benefit from Expanding beyond simple working memory tasks in an attempt to characterize the specificity (or generalizability) of functional deficits. We strongly agree with this sentiment, and feel this preliminary work is a significant contribution to the field as we have developed fMRI-compatible computerized NP tasks similar to those used clinically. The development of computerized NP tests has evolved from traditional paper and pencil tests adopted from the most sensitive tests in the mTBI literature. Currently, all of the computerized NP test batteries attempt to evaluate various domains of cognitive functioning quickly and efficiently, using a variety of timed tasks (42–46). Domains of testing include working memory, attention, concentration, information processing, reaction time, and short-term verbal and non-verbal memory abilities. Central to the use of computerized NP assessment is the tenet that each specific test assesses a targeted cognitive function; however, the evidence for such claims is less convincing. The findings from these two cases are promising, as distinct activation patterns were associated with purported cognitive domains for each of the tests (see Figures 1–3). It is imperative that future large-scale studies demonstrate explicitly that performance of the various elements of such a test battery is correlated with particular activation states in specific regions of the brain in athletes with concussion as well as uninjured athletes.

Despite the similarity in the general regions of activation identified in both athletes, the two reported cases highlight some important empirical considerations moving forward. First, the severity of symptoms was mild for both athletes, yet they each displayed distinctly different patterns of BOLD signal activation (Figures 1–3). Previous studies that have examined BOLD responses and symptoms associated with concussion (31, 34, 46) have identified a general trend where more severe symptoms are associated with a reduced BOLD signal. However, this was not the case in the present study. Second, in studies that have reported similar performance between concussed athletes and uninjured controls, athletes with concussion have shown greater BOLD activity than controls (29, 31, 34). This pattern of activity, in the absence of performance declines, may reflect the compensatory recruitment of additional brain areas to maintain the same cognitive function. In the present study, the greater BOLD activation (see Case 1 JZ in Figures 1–3) was identified in the athlete presenting with better performance on the cognitive tasks (Table 2), and in brain areas that would be unexpected given the task requirements; therefore, compensatory performance behavior in this case is plausible. On the other hand, as illustrated in Figure 1, the BOLD activation pattern of Case 2 (HC) was similar to previous reports of reduced BOLD activity in the symptomatic phase. However, HC reported few symptoms 2 days prior to fMRI. Collectively, based on lower cognitive scores and reduced BOLD signal, we question the reliability of symptom reporting in this particular case. The present study was limited to data of individual subjects, and future studies will be strengthened significantly by investigating a cohort of athletes providing group BOLD activation maps, with the acquisition of outcome measures (symptom checklist and NP performance) at the same time as fMRI. A well-matched control group will also be essential to characterize the normal patterns of BOLD activation, as well as test–retest reliability, for specific clinical NP tasks. At the same time, future studies should continue to explore the development of appropriate NP tasks suitable for the fMRI environment and clinically relevant to the population of the interest. Studies including these elements will be in a position to address the important outstanding question of how the brain changes throughout the recovery process and RTP.

**Table 2:** **Performance on computerized assessments during fMRI**.

	Case 1 – JZ	Case 2 – HC
**SIMPLE REACTION TASK**		
Mean reaction time (ms)	330	570
Accuracy (% correct)	100	100
**SPATIAL PROCESSING TASK**		
Mean reaction time (ms)	1580	1870
Accuracy (% correct)	93	83
**MATCH-TO-SAMPLE TASK**		
Mean reaction time (ms)	1380	1800
Accuracy (% correct)	100	84

For the cases reviewed, fMRI was completed during the initial stages of the recovery process (<10 days following concussion), and as discussed above we question the reliability of symptom reporting based on BOLD activation and cognitive performance. In the Chen and colleagues study (29), one athlete was re-tested when their post-concussive symptoms resolved. The athlete showed striking changes in activation patterns, from a widespread distribution in the acute phase to an activation pattern similar to that of the control subjects during the post-RTP phase. In future studies, the ability to document brain changes associated with symptom recovery, NP performance, and the clinical determination of RTP will extend our understanding of the pathophysiology of concussion. If we find that abnormal brain states persist beyond clinically determined readiness for RTP, then it may call into question the currently accepted clinical RTP guidelines.

## Concluding Remarks

Future research is required to better understand the utility of NP tests in concussion management and how the tests relate to brain recovery. We believe that advanced neuroimaging techniques, such as those adopted to report on two case studies in the present article, have the potential to advance our understanding and substantially influence the clinical management of sport-related concussion.

## Author Contributions

Michael G. Hutchison, Tom A. Schweizer, Simon J. Graham were involved in all aspects of the study including the study design, data analysis, data interpretation, and manuscript preparation. Paul Comper is the clinic’s consulting neuropsychologist and was involved in the development of case reports and manuscript preparation. Fred Tam was involved in fMRI test development, post processing of magnetic resonance images, data analysis, and manuscript preparation. All authors approved of the final version of the manuscript.

## Conflict of Interest Statement

The authors declare that the research was conducted in the absence of any commercial or financial relationships that could be construed as a potential conflict of interest.
